# Decoding Heterogeneity in Data-Driven Self-Monitoring Adherence Trajectories in Digital Lifestyle Interventions for Weight Loss: A Qualitative Study

**DOI:** 10.21203/rs.3.rs-3854650/v1

**Published:** 2024-01-19

**Authors:** Shiyu Li, Yan Du, Christiane Meireles, Dan Song, Kumar Sharma, Zenong Yin, Bradley Brimhall, Jing Wang

**Affiliations:** Department of Kinesiology, Pennsylvania State University; School of Nursing, UT Health San Antonio; School of Nursing, UT Health San Antonio; College of Nursing, Florida State University; Long School of Medicine, UT Health San Antonio; Department of Public Health, The University of Texas at San Antonio; Long School of Medicine, UT Health San Antonio; College of Nursing, Florida State University

**Keywords:** data science, qualitative research, lifestyle interventions, obesity, self-monitoring, adherence

## Abstract

**Background:**

Data-driven trajectory modeling is a promising approach for identifying meaningful participant subgroups with various self-monitoring (SM) responses in digital lifestyle interventions. However, there is limited research investigating factors that underlie different subgroups. This qualitative study aimed to investigate factors contributing to participant subgroups with distinct SM trajectory in a digital lifestyle intervention over 6 months.

**Methods:**

Data were collected from a subset of participants (n = 20) in a 6-month digital lifestyle intervention. Participants were classified into Lower SM Group (n = 10) or a Higher SM (n = 10) subgroup based on their SM adherence trajectories over 6 months. Qualitative data were obtained from semi-structured interviews conducted at 3 months. Data were thematically analyzed using a constant comparative approach.

**Results:**

Participants were middle-aged (52.9 ± 10.2 years), mostly female (65%), and of Hispanic ethnicity (55%). Four major themes with emerged from the thematic analysis: Acceptance towards SM Technologies, Perceived SM Benefits, Perceived SM Barriers, and Responses When Facing SM Barriers. Participants across both subgroups perceived SM as positive feedback, aiding in diet and physical activity behavior changes. Both groups cited individual and technical barriers to SM, including forgetfulness, the burdensome SM process, and inaccuracy. The Higher SM Group displayed positive problem-solving skills that helped them overcome the SM barriers. In contrast, some in the Lower SM Group felt discouraged from SM. Both subgroups found diet SM particularly challenging, especially due to technical issues such as the inaccurate food database, the time-consuming food entry process in the Fitbit app.

**Conclusions:**

This study complements findings from our previous quantitative research, which used data-drive trajectory modeling approach to identify distinct participant subgroups in a digital lifestyle based on individuals’ 6-month SM adherence trajectories. Our results highlight the potential of enhancing action planning problem solving skills to improve SM adherence in the Lower SM Group. Our findings also emphasize the necessity of addressing the technical issues associated with current diet SM approaches. Overall, findings from our study may inform the development of practical SM improvement strategies in future digital lifestyle interventions.

**Trial registration:**

The study was pre-registered at ClinicalTrials.gov (NCT05071287) on April 30, 2022.

## BACKGROUND

More than 40% of American adults are obese (1). Obesity is associated with a range of major comorbidities and imposes a significant economic burden on the society. In 2018, the total direct medical cost of obesity among adults in the United States exceeded $260 billion (2). Behavioral lifestyle interventions have been recommended as front-line treatment strategies for adults with overweight or obesity (3). Self-monitoring (SM) is a major component in these lifestyle interventions to support diet and physical activity (PA) behavior improvements. According to the Theory of Self-Regulation, by deliberately observing and recording one’s behavior and health, SM raises one’s awareness and enables necessary behavioral adjustments (4, 5). However, SM adherence declines over time, which is consistently reported to be associated with poorer weight loss outcomes (5, 6). Thus, optimizing SM components is crucial to enhance the effectiveness of behavioral lifestyle interventions for obesity management.

Several studies have developed and examined strategies to improve SM adherence, but they often overlooked individual differences in SM behaviors (7). In a recent Delphi study by Krukowski and others (8), experts reached a consensus recommending abbreviated SM strategies to improve adherence, including (1) weight SM only, (2) simplifying diet SM to less frequent and reporting calorie-dense foods only, and (3) reducing the frequency of detailed diet SM. These recommendations were formulated based on group-level evidence, assuming that all individuals experience declined SM for the same underlying reason. However, a recent systematic review identified various reasons contributing to declined use of physical activity SM, including data inaccuracy, privacy concerns, inconvenience of use, loss of SM motivation, etc. (9). These reasons suggested that there is no “one-size-fits-all” solution for sustaining long-term SM adherence. Developing and prescribing SM strategies that account for individual differences in SM behaviors is essential.

Data-driven statistical models have been employed to identify participant subgroups with distinct SM patterns. In our recent study, we used group-based multi-trajectory modeling to estimate adherence patterns to SM of diet, physical activity, and weight among adults with overweight or obesity in a 6-month digital lifestyle intervention (10). Two participant subgroups emerged: the “Lower SM Group”, with low and rapidly declining SM adherence for all SM targets, and the “Higher SM Group”, with moderate and declining diet and weight SM and high physical activity SM. Starting from week 2, the Lower SM Group showed significantly lower level of SM across all SM targets. By 6 months, only the Higher SM Group reported significant weight loss and HbA1c control. While our quantitative findings underscore the utility of data-driven trajectory modeling approaches for identifying unobserved population subgroups (11), the clinical relevance of these subgroups remains a question (12). In this context, we must further probe: Do individuals within the same subgroup truly share similarities? Are individuals from different subgroups really different? And what drives the differences between subgroups?

To facilitate the translation of findings from data-driven models into clinical practice, we analyzed qualitative data collected from semi-structured interviews at 3 months regarding participants’ experiences and perceptions with SM using the same sample as the quantitative study. Qualitative data is invaluable as it offers deep insights into individual behaviors, experiences, perspectives, and surrounding contexts (13). By exploring factors that facilitated or hindered SM adherence, we aimed to inform SM improvement strategies in future digital lifestyle interventions for obesity management.

## METHODS

### Study Design and Sample

This study used data collected from participants in a pilot randomized controlled trial. The parent trial was a 6-month randomized controlled trial examining the effect of a digital lifestyle intervention in overweight/obese adults. Enrolled participants were adults (≥ 18 years of age) who were overweight or obese (BMI ≥ 25 kg/m^2^), with or without diagnosis of type 2 diabetes, and with or without evidence of chronic kidney diseases. Participants were randomly assigned to either a low-fat low-calorie diet or a ketogenic diet. Digital lifestyle interventions were offered during the trial, including digital education, individual counseling sessions, and personalized feedback. All participants were encouraged to SM diet, physical activity, and weight daily over 6 months. All study procedures were approved by the Institutional Review Board of [Blinded for Review]. Further details of the trial have been reported elsewhere and only study procedures of relevance to this study’s aim are reported here (14).

**Figure 1:**
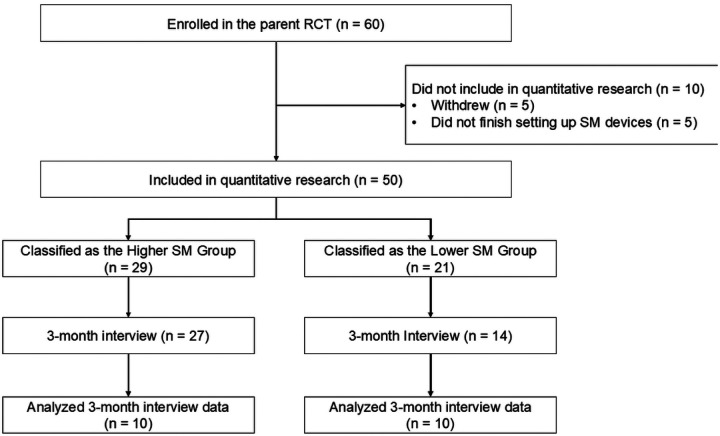
Sampling for the qualitative study. (RCT: randomized controlled trial; SM: self-monitoring)

During the parent trial, 50 participants did not withdraw and provided at least one SM data point over 6 months. These participants were grouped into either a “Higher SM” subgroup or a “Lower SM” subgroup, based on their 6-month SM adherence trajectories. All participants were invited for a three-month individual interview. For this qualitative study, we aimed for equal sample sizes for each of two participant subgroups (Fig. 1).

### Data Collection

A semi-structured interview guide consisting of five open-ended questions with optional probing was developed by members of our research team (SL, YD, CM, JW) (Table 1). The team was composed of nursing scientists with extensive experience in digital health and qualitative research, research coordinators experienced in patient communication and qualitative research, and a registered dietitian. The guide was developed to explore SM perceptions and experiences, facilitators and barriers to SM, and suggestions for SM strategies.

Each interview lasted for 15 to 30 minutes. For the ketogenic diet group, a dietitian conducted the interviews, and a research coordinator took detailed notes during the interview. For the low-fat low-calorie diet group, a research coordinator conducted the interviews, and a research assistant took detailed notes. Notetakers transcribed statements from the interviewees as much as possible rather than summarizing answers. All interviews were conducted virtually on Zoom (Zoom Video Communications Inc., USA). All interview notes were deidentified and imported into Microsoft Excel (Microsoft Corporation, USA) for analysis.

### Data Analysis

Thematic analysis, with no predetermined theory, structure, or framework, was conducted across participant subgroups “identify, explore, explain, and compare” themes expressed by subgroups about SM (15). We followed a deductive approach in which themes were derived from the dataset itself (16). First, two researchers (SL and DS) familiarized with the data through reading the interview notes multiple times. Second, two researchers each inductively coded data to generate a codebook and grouped codes into themes across trajectory subgroups. Frequent repetition of codes occurred after analyzing around 10 interview notes for each trajectory group. The research team decided data saturation was achieved, and thus, a total of 20 interview notes were used to generate codebooks and themes. Third, SL and DS gathered to compare codebooks, coded texts, and themes. Discrepancies were reconciled through discussion between the two researchers until a consensus was reached. A senior qualitative researcher (YD) was brought for consultation throughout the process as needed.

## RESULTS

Baseline characteristics of the sample are presented in Table 2, stratified by SM trajectory subgroup. Participants were middle-aged (52.9 ± 10.2 years), mostly female (65%), and of Hispanic ethnicity (55%).

Qualitative analysis of individual interview notes revealed the following major themes emerged from the interview data: (1) Acceptance towards SM Technologies, (2) Perceived SM Benefits, (3) Perceived SM Barriers, and (4) Responses When Facing SM Barriers. These major themes were shared between the Lower and Higher SM groups, but the codes within these themes differed slightly between the two subgroups.

### Theme 1. Acceptance towards SM Technologies

This theme comprises the features and functionalities of SM technologies that gained acceptance among participants in both subgroups and might facilitate SM technology utilization.

Participants in both groups (Lower SM: 2, Higher SM: 4) expressed their acceptance towards SM technologies in terms of their accuracy.

“*Has compared with an exercise activity app that he has been using for a long time now, and finds that the Fitbit is similar to the results from the app*.”

[Higher SM, Male, 44 years old]

“Finds it (Fitbit food log) to be accurate compared to the MyFitnessPal app. Finds the apps to be the same.”

[Lower SM, Male, 41 years old]

“Scale works well and is accurate”

[Lower SM, Male, 43 years old]

Each SM subgroup had one participant who mentioned the ease of use associated with the automatic syncing function of the weight scale.

“Scale – simple and easy to use and syncs to phone very easily”

[Higher SM, Male, 51 years old]

“Weight scale – likes it, automatically connects over and finds it to be the easiest one”

[Lower SM, Female, 55 years old]

Two individuals in the Higher SM group also stated a preference for non-study-related features of SM technology, such as body fat percentage, daily temperature readings on the weight scale, and sleep pattern monitoring.

### Theme 2 Perceived SM Benefits

Interview notes from participants in both subgroups regarding the perceived SM benefits included two common key codes: (1) SM offered positive feedback and (2) SM facilitated health behavior change.

Participants in both subgroups (Lower SM: 4, Higher SM: 2) perceived the fitness tracker and the weight scale as sources of positive feedback, motivating participants to stay committed and continue their efforts.

“FIt tells the steps she has been taking, likes to see it during exercise. Is working good, no problems with it”

[Lower SM, Female, 53 years old]

“Weight scale – easy to use, will show you how much you have lost which is a good incentive. Finds it to be efficient.”

[Higher SM, Female, 67 years old]

Participants in both subgroups (Lower SM: 1, Higher SM: 4) also recognized the facilitating role of diet SM for diet adherence.

“The fitbit food logs has helped the most (on following the diet), seeing if it was higher or lower in calories in general. Also helped to prepare her own portions (grams and oz and serving size). Understanding sizes and what that means calorically, and then putting it in on the Fitbit.”

[Lower SM, Female, 48 years old]

“Using the Fitbit has helped a lot (on following the diet), being able to see and track foods and choices.”

[Higher SM, Male, 53 years old]

In addition to perceiving SM as positive feedback, a subset of participants in the Higher SM group (n = 3) underlined the sense of enjoyment they experienced from observing SM results.

“Likes using it, can see progress throughout the day can see the 10,000 step goal”

[Higher SM, Male, 53 years old]

“loves the scale, thinks it is amazing, likes the tracking of ups and downs.

[Higher SM, Female, 66 years old]

Furthermore, SM has become part of the daily habit for some participants (n = 3) in the higher SM group.

### Theme 3. Perceived SM Barriers

Barriers to SM can be categorized into individual-level and technical-level. At individual-level, both subgroups (Lower SM: 1, High SM: 2) reported occasions forgetting to SM.

“Forgets to do it a lot. Does not like to do it before in case gets distracted or called for work and doesn’t end up eating.”

[Lower SM, Male, 41 years old]

“(Self-monitoring) has been challenging, forgets sometimes.”

[Higher SM, Female, 46 years old]

Some (n = 2) in the Lower SM group also attributed their lack of SM to a busy schedule.

“gets busy with work. Then much time had passed and continued to get busy and not log any food.”

[Lower SM, Female, 31 years old]

Individual-level barriers to SM that are specific to the Lower SM group include a lack of knowledge about how to SM (n = 4).

“Has not been tracking food. (Hope for the study team to) do a video of the food logging and give out.

[Lower SM, Female, 66 years]

Technical level barriers that hindered both subgroups from SM included inaccuracy, burden of diet SM, lack of customization, preference for non-study provided SM technologies, and lack of data integration

Inaccuracy emerged as a major concern for participants in both subgroups (Lower SM: 3, Higher SM: 8) that might discourage them from continuing with SM.

“When putting in foods like salads, shows much more carbs than is in the food actually.”

[Higher SM, Male, 50 years old]

“Too much walking logged for what is actually happening. Couple hundred steps off usually. Does autotrack, but makes it seem like it is sprinting occasionally. Hits steps when driving sometimes.”

[Lower SM, Male, 47 years old]

Diet SM was regarded as time-consuming and burdensome by participants in both subgroups (Lower SM: 6, Higher SM: 3).

“takes up a lot of time to input all of the food. Has not put in for about two weeks.”

[Lower SM, Female, 53 years old]

“Does not like using the app. Has to manually input a lot of things in there, especially if going out to eat. Has to ask the place for nutrients and then manually input it.”

[Higher SM, Female, 49 years old]

Many participants also mentioned the lack of customization in SM devices (Lower SM: 6, Higher SM: 5), particularly the Fitbit food logging feature and its extensive and confusing food database.

“Two of the same products (strawberries) will come up as different calories depending on the store buying from in Fitbit, even though the product is the same.”

[Higher SM, Female, 49 years]

“the calorie range varies even within food item.”

[Lower SM, Female, 57 years old]

Participants in both subgroups emphasized that logging homemade foods on Fitbit was particularly challenging, noting that it was generally more cumbersome to log homemade meals compared to eating out.

“Many random foods that come up and fast-foods come up first. When doing home made foods, it is hard to know for example: if meatloaf made at home is similar to a meatloaf out to eat. When a food item does come up, there are several options which are not the basic food and rather from a restaurant which may not be accurate.”

[Lower SM, Male, 42 years old]

“Is simple when doing or scanning a barcode, but when making meals has to enter each item individually which is more difficult.”

[Higher SM, Male, 53 years old]

Participants in both groups (Lower SM: 1, Higher SM: 2) expressed a desire for an “all-in-one” platform to reduce their burden associated with using multiple SM technologies and mobile applications.

“Thinks some of it should be condensed. Feels like you have to bounce from app to app. One device or app to do everything.”

[Higher SM, Female 49 years old]

“Switching to a new app like MyFitness pal, and integrating apps together like the apple phones can sometimes do.”

[Lower SM, Male, 43 years old]

Additionally, participants in the Lower SM (n = 3) emphasized their preference for personally owned SM devices over study-provided ones, citing reasons related to usability and functionalities.

“MyFitnessPal is more user friendly. Hard to find things in Fitbit and log them.”

[LowerSM, Male, 43 years old]

### Theme 4. Responses When Facing SM Barriers

A major difference between the two subgroups was how they reacted to SM barriers. Notably, due to above-mentioned barriers, some participants (n = 3) in the lower SM group reported a shift in their attitude from initially positive to negative after SM for a few weeks.

“It was good at first, it was exciting in the beginning. After the first few weeks, it was hard to keep up with and often forgets to log food or do the blood sugar readings.”

[Lower SM, Female, 53 years old]

However, in the higher SM group, most participants (n = 8) self-initiated positive problem-solving strategies and action planning skills to overcome barriers and continue with SM.

“Scale is connected to Wi-Fi, so whenever the Wi-Fi is changed (every few days depending on if staying with significant other), the Wi-Fi needs to be changed again. Has found a way around this by adding it as a new item in the app and it keeps the old data in there.”

[Higher SM, Male, 44 years old]

For example, many participants in the Higher SM Group (n = 8) described writing things down as a good approach for recalling their daily meals and found it improved adherence to diet SM.

“Carries a written log and then goes back and puts them in later into the Fitbit”

[Higher SM, Male, 50 years old]

Planning and logging all meals at the beginning of the day before eating was also mentioned as an effective strategy to promote adherence to diet SM (n = 2).

“(Logging foods) At the beginning of the day if knowing what will be eaten during the day, and then will add other items right away to not forget.”

[Higher SM, Female, 46 years old]

## DISCUSSION

In a previous quantitative analysis, we used data-driven trajectory modeling approach to identify distinct participant subgroups in a digital lifestyle intervention based on their 6-month SM adherence trajectories. This current qualitative research complements the quantitative study. Findings from this study revealed factors that led to differences in SM experiences and behaviors among the subgroups. Notably, participants in the Higher SM Group commonly described their adoption of positive problem-solving strategies to overcome SM barriers. On the other hand, participant from both subgroups consistently reported technical difficulties, especially with the burdensome diet SM component. This emphasizes the necessity of refining the technical aspects of diet SM. Taken together, our findings have the potential to inform the development of practical SM improvement strategies in future digital lifestyle interventions for obesity management.

Despite facing similar barriers to SM, the Higher SM group used more problem-solving skills to overcome these barriers effectively, whereas the Lower SM group tended to feel discouraged. Problem solving is a core skill to support chronic disease self-management (17). Previous research found that individuals with stronger problem-solving skills had better adherence to diet SM and weight loss in a 6-month behavioral lifestyle intervention (18). However, behavioral lifestyle interventions alone are insufficient to impact one’s problem-solving ability. According to Yu et al., the problem-solving ability of individuals participating a behavioral lifestyle intervention for weight loss remained stable over 12 months (19). Thus, our findings suggested that providing supplemental problem-solving therapy to address SM barriers for participants with lower level of SM adherence during the early phase of an intervention could be beneficial.

Both subgroups perceived SM as a source of positive feedback and a facilitator for behavior changes. Notably, the high SM group reported feeling enjoyment from the positive feedback, with some even forming habits around SM. This suggests that autonomous motivation could be a driving cause underlying their higher level of SM adherence (20). According to the Self-Determination Theory, feeling a sense of enjoyment during behavior engagement indicates autonomous motivation, a characteristic consistently linked to long-term behavior change (21). Previous research has emphasized the importance of positive feedback in satisfying basic psychological needs for competence and autonomy, both of which are necessary for building autonomous motivation (22). Furthermore, research has associated autonomous motivation with habit formation, emphasizing the importance of autonomous motivation in forming habitual behaviors (23). Taken together, our study suggests a potential mechanism underlying SM habit formation. Receiving constant positive feedback from SM technologies increases individuals’ perceived competence in adopting healthier lifestyle and SM, hence enhancing their autonomous motivation, resulting in sustained SM adherence and SM habit formation. In other words, positive feedback from SM technologies may promote long-term SM adherence and SM habit formation. Thus, our findings highlight the need for intensive intervention strategies to initiate behavior change, allow individuals to receive positive feedback from SM technologies and thereby support the formation of SM habits.

Despite different brands and types, SM technologies are often grouped together in the relevant literature under terms such as “digital food diary,” “fitness tracker,” and “smart scale” (24). Interestingly, many in the Lower SM group favored certain SM technology brands over those provided by the study team due to perceived advantages in accuracy, usability, ease of use, and aesthetics. Given that these SM technologies shared similar characteristics (e.g., SM, positive feedback, goal setting), future study should investigate the potential moderating role of SM technology brands on SM adherence (25). Furthermore, our findings also suggest that a lower level of adherence to SM may not always imply disengagement from SM. Instead, it could be the result of an individual switching to alternate SM technologies after being dissatisfied with the ones offered by the study team. In such situations, it is important for the interventionist to assess the individual’s needs for app features and assist him/her in selecting SM technologies that fit best with their needs and preferences in order to prevent him/her from disengaged SM (26).

Our findings revealed several technical aspects that can be improved to reduce the burden associated with diet SM. One major source of burden was the time-consuming manual entry process for homemade meals compared to restaurant meals. Embedding computer-vision algorithms into diet SM apps holds great promise in simplifying the process of adding new homemade meals to the food database (27). Previous research has revealed that food identification apps using computer vision algorithms may easily and accurately capture detailed information about food products (28). Another commonly reported burden of diet SM was the extensive yet confusing built-in food database. Similar to previous research, common issues associated with the food database include the inaccurate nutritional information, the presence of multiple entries for the same food item, and the requirement for precise search terms to locate desired food products (29, 30, 31). Thus, additional effort is required on the part of app developers to improve the food database and assure the quality and accuracy of information in the food database. Another source of burden was the need to bounce back and forth between different apps to complete SM.

Our findings, similar to previous studies, emphasized the necessity for all-in-one SM apps to reduce SM burden (32).

There are several limitations to be noted. First, the qualitative data was collected at 3 months, which was a relatively short period of time given that weight loss and maintenance require long-term commitment. Future studies are needed to explore how perceptions of SM change over time to gain a comprehensive understanding of SM behavior. Second, the sample was derived from a subset of the parent trial participants who sustained engagement for 3 months. Consequently, our findings might reflect a biased population with greater commitment to SM and may not be generalizable to the entire population. Additionally, we relied on interview notes in the qualitative data analysis. Although the research coordinators made efforts to transcribe the conversations accurately, there could be missing or potentially biased information in these notes.

## CONCLUSION

Using data-driven trajectory modeling approach, our previous quantitative research identified participant subgroups with distinct SM adherence trajectories over a 6-month behavioral lifestyle intervention. This qualitative study offered a comprehensive and context-specific understanding of SM experiences across these subgroups. We identified key SM facilitators and barriers shared by both subgroups. Further, participants with stronger positive problem-solving and action planning skills were more likely to overcome SM barriers and SM more frequently. Taken together, findings from our study can aid in the development of practical SM improvement strategies in future digital lifestyle interventions for obesity management.

## Figures and Tables

**Table 1:** Semi-structured interview questions.

**1. How has your experience been in using SM devices in general?**
2. How has your experience been with food logging (i.e., any difficulties, inaccuracies, pros of food logging observed)? If no self-monitoring of food logging - do you know why you need to monitor food?
3. At what time/when do you enter your food logs?
4. What suggestions you can give to improve the food logging process?
5. How has your experience been with using the Fitbit as an activity tracker?

**Table 2:** Baseline characteristics of the qualitative sample (n = 20).

	Total (n = 20)	Lower SM (n = 10)	Higher SM (n = 10)
Age	52.9 ± 10.2	51.2 ± 11.1	54.5 ± 9.5
Female	13 (65%)	7 (70%)	6 (60%)
Hispanic	11 (55%)	5 (50%)	6 (60%)
Annual Household Income $80,000 or Higher	7 (35%)	4 (40%)	3 (30%)
College Degree or Higher	9 (45%)	4 (40%)	5 (50%)
Type 2 Diabetes	7 (35%)	2 (20%)	5 (50%)
Body Weight (kg)	95.3 ± 19.0	90.1 ± 14.6	100.5 ± 22.1
HbA1c (%)	5.7 ± 0.8	5.6 ± 0.5	5.8 ± 0.9

## Data Availability

The datasets used and/or analyzed during the current study are available from the corresponding author on reasonable request.

## Abbreviations

PA: Physical Activity
SM: Self-monitoring
RCT: Randomized Controlled Trial
